# Functions of Kinesin Superfamily Proteins in Neuroreceptor Trafficking

**DOI:** 10.1155/2015/639301

**Published:** 2015-05-17

**Authors:** Na Wang, Junyu Xu

**Affiliations:** Department of Neurobiology, Key Laboratory of Medical Neurobiology (Ministry of Health of China), Collaborative Innovation Center for Brain Science, Zhejiang University School of Medicine, Hangzhou, Zhejiang 310058, China

## Abstract

Synaptic plasticity is widely regarded as the cellular basis of learning and memory. Understanding the molecular mechanism of synaptic plasticity has been one of center pieces of neuroscience research for more than three decades. It has been well known that the trafficking of *α*-amino-3-hydroxy-5-methylisoxazoloe-4-propionic acid- (AMPA-) type, N-methyl-D-aspartate- (NMDA-) type glutamate receptors to and from synapses is a key molecular event underlying many forms of synaptic plasticity. Kainate receptors are another type of glutamate receptors playing important roles in synaptic transmission. In addition, GABA receptors also play important roles in modulating the synaptic plasticity. Kinesin superfamily proteins (also known as KIFs) transport various cargos in both anterograde and retrograde directions through the interaction with different adaptor proteins. Recent studies indicate that KIFs regulate the trafficking of NMDA receptors, AMPA receptors, kainate receptors, and GABA receptors and thus play important roles in neuronal activity. Here we review the essential functions of KIFs in the trafficking of neuroreceptor and synaptic plasticity.

## 1. Introduction

For more than three decades, synaptic plasticity holds the greatest promise for uncovering a key event in neuroscience research: the cellular basis of learning and memory in the mammalian brain. It has been shown that the *α*-amino-3-hydroxy-5-methylisoxazoloe-4-propionic acid- (AMPAR-) type and N-methyl-D-aspartate- (NMDAR-) type glutamate receptors are critical for the induction and expression of long-term potentiation (LTP) and long-term depression (LTD) (see [1–6] for reviews). AMPARs and NMDARs are mainly concentrated at the postsynaptic site of excitatory synapses [7–9]. Different working models have been offered in understanding how AMPARs are recruited to the synapse during LTP (see [10] for review). Among which, scaffold proteins and adaptor proteins are involved in regulating AMPARs phosphorylation and membrane trafficking. Many cytoskeleton or synaptic structural proteins locally interact with these receptors and organize their localization in postsynaptic sites through the PDZ (PSD-95/Dlg/ZO-1) domain interaction. PSD-95, as well as other synaptic proteins, binds to the C-terminus of the receptors through its PDZ domains [11]. Overexpression of PSD-95 increases the number of synapse expressing AMPARs [12, 13]. The NMDARs subunit GluN2B binds directly to the two N-terminal PDZ domains of PSD-95, and GluN2B colocalizes with PSD-95 in cultured rat hippocampal neurons. This interaction serves to anchor NMDA receptors to the submembrane cytoskeleton [14, 15]. PICK1, another PDZ-containing protein, interacts with AMPAR subunits GluA2, GluA3, and GluA4c [16, 17]. PICK1-GluA2 interaction is involved in the removal of GluA2 from plasma membrane during the induction of LTD in hippocampus [5–7] and cerebellum [16, 17]. PICK1 is involved in the constitutive trafficking of AMPARs in basal conditions [6] and recycling of internalized AMPARs back to plasma membrane [10–12] and is critical for hippocampal LTP as well [13]. In addition, NMDAR-dependent long-term synaptic plasticity is related to PICK1 [18]. GRIP is another adaptor protein that links AMPARs to other proteins and it is critical for the clustering of AMPARs at excitatory synapses in the brain [19, 20]. The turnover between GRIP and PICK1 for AMPARs binding has been reported to be critical for endocytosis of AMPARs during LTD [21–23].

Other than PDZ domain containing proteins, the kinesin superfamily proteins (KIFs), are also related to synaptic plasticity by transporting receptors in-between different compartments of neuron. KIFs are microtubule-dependent molecular motors. KIFs can transport various types of cargo, including organelles, synaptic vesicle precursors, neurotransmitter receptors, cell signaling molecules, cell adhesion molecules, and mRNAs in neurons of mammalian nervous system [24, 25]. There are 45 kinesin superfamily genes in the mouse genome, which are classified into 15 families [26]. Up till now, no more than 10 kinesin superfamily proteins are reported to transport the ionic neurotransmitter receptors and are involved in synaptic plasticity.

In addition to AMPARs and NMDARs, kainate receptors (KARs) are another type of ionotropic glutamate receptors [27], which play a critical role in synaptic transmission [28] and brain disorders including schizophrenia [29] and autism [30]. Recently, the localization of GluR5, one of KAR subunits, was proved to be related with KIFs [31].

GABA receptors also participate in modulating synaptic plasticity. There were two main classes of GABA receptors, the ionotropic type A GABA receptors (GABA_A_Rs) and the G-protein-coupled metabotropic type B GABA receptors (GABA_B_Rs). GABA_A_Rs are one of the main subtypes of GABA receptors related to LTP; it can ensure that NMDARs are activated mainly during high-frequency synaptic transmission [6]. The activation of GABA_A_Rs and GABA_B_Rs receptors together limits the synaptic activation of NMDARs during low-frequency activation. During high-frequency activation, GABA receptors depress its own release and permit sufficient NMDAR activation for the induction of LTP [32]. KIFs have as well been identified to bind with GABA receptors and help modulating the synaptic plasticity [33–38].

The metabotropic glutamate receptors (mGluR) are well-reported for the induction of LTD in CNS (see [1] for review). However, currently there is no literature about the kinesin-regulated mGluR trafficking.

In this review, we will mainly focus on the KIFs related with AMPARs, NMDARs, KARs, and GABARs trafficking and look into the details of their regulatory mechanism, including their specific cargo receptors, the adaptor proteins in KIF-containing vesicles, and the involvement in synaptic plasticity.

## 2. Roles of Kinesin Superfamily Proteins in AMPA Receptors Trafficking

It has been widely accepted that the alteration in AMPARs number is one of the expression mechanisms for LTP and LTD [39, 40]. Cumulative evidence demonstrated that the trafficking of AMPARs away from or to the membrane is involved in synaptic plasticity [41]. Various kinases, such as CaMKII [41], PI3K [42], and PKC [2], have been reported to modulate AMPARs trafficking during synaptic plasticity through receptor phosphorylation.

Interestingly, electrophysiological studies indicated that AMPAR-mediated synaptic transmission is dependent on kinesin superfamily proteins. AMPAR-mediated currents were decreased by the application of anti-kinesin and anti-dynein antibodies [43]. KIF5, firstly identified and characterized in neurons [44], belongs to kinesin 1 family [45] and is found to be expressed in the murine CNS [46]. KIF5 is expressed in various animal species and is the mouse homologue of the ubiquitous human kinesin heavy chain (KHC) [47]. Gene duplication makes three subtypes of KIF5: KIF5A, KIF5B, and KIF5C [44, 48, 49]. Distinct from ubiquitously expressed KIF5B, KIF5A, and KIF5C are neuron-specific [50, 51]. In neurons, KIF5 was found to be related with AMPARs trafficking. The AMPAR subunit-GluA2-interacting protein (GRIP1) [19] was found interacting with KIF5B* in vivo* and with KIF5A-C* in vitro* [52]. KIF5B is required for the normal localization of GRIP1 since in cultured neurons of KIF5B knockout mice, GRIP1 was concentrated in the center rather than in the periphery region [52, 53]. Further investigation found that GRIP1, GluA2, and KIF5 form complex* in vivo*, and GRIP1 could recruit KIF5 to the soma and dendrites in neurons through the kinesin binding site between PDZ6 and PDZ7 domain. The KIF5-GRIP1 complex was suggested to transport AMPARs as overexpression of dominant negative form of KIF5 (KHCCBD) reduced the amount of GRIP1 and GluA2 in synapses [52]. A recent paper shows that N-Cadherin was identified binding with the PDZ domain of GRIP1, using yeast two-hybrid system [54]. KIF5, GRIP, N-Cadherin, and GluA2 are found to form a large complex, and coexist in vesicles, and are colocalized along dendrites [54]. Live imaging of the moving vesicles revealed the comigration of N-Cadherin and GluA2, which could be effectively disrupted by overexpression of the KIF5C mutant lacking the motor domain (GFP-KIF5CΔMD) [54]. Moreover, KIF5CΔMD reduced the surface expression level of GluA2 [54], suggesting KIF5C mediates long-distance transport of GluA2-containing vesicles. It is possible that the KIF5-GRIP1-N-cadherin complex may further participate in synaptic plasticity such as LTP or LTD by regulating the trafficking and surface level of GluA2.

In addition, EphB receptors were reported to interact with AMPARs regulatory proteins GRIP1 and PICK1 [55] and also colocalize with NMDARs to their receptors function [56]. In addition to AMPARs, KIF5-GRIP1 interaction was also found to modulate EphB2 receptors trafficking and dendritic growth [57]. When the KIF5-GRIP1 interaction is disrupted, the trafficking of EphB receptor and dendritic morphology can be impaired [57]. Therefore, KIF5 might also regulates synaptic plasticity by transporting EphB receptors.

In* C. elegans*, KIF5 can modify the delivery, removal, and redistribution of synaptic AMPARs. In unc116/KIF5 mutants, GLR-1 surface expression is obviously increased and glutamate-gated currents are decreased [58].

In addition to KIF5, KIF1A was found to bind with liprin-*α* directly. The C terminus of liprin-*α* interacts with GRIP [59]. Both of KIF1A and liprin-*α* distribute in dendrites and axons in rat brain and cultured hippocampal neurons. KIF1A was found to form a complex with AMPA receptor-GRIP complex through liprin-*α* [60]. Though there has not been direct evidence showing whether KIF1A truly regulates AMPARs trafficking or not, it is highly possible that KIF1A may be also part of the GRIP-mediated AMPARs trafficking machinery.

The kinesin-3 family motor KLP-4/KIF13 can regulate trafficking of GLR-1 glutamate receptors in the ventral nerve cord of* C. elegans *[61]. It can also modulate GLR-1 dependent locomotion behavior [61]. In klp-4 mutant worms, GLR-1 accumulates in the cell bodies because of decreased degradation [61].

## 3. Roles of Kinesin Superfamily Proteins in NMDA Receptors Trafficking

NMDARs are the known triggers for the induction of LTP and LTD. The expression of NMDARs at synapse can be modulated by several phosphorylation-involved pathways, such as the activation of PKA and PKC [62–64], Src [65], CaMKII [66], and CDK5 [67]. GluN1 and GluN2 subunits of NMDARs are also trafficked within dendrites, which are related to microtubules [68, 69]. According to the electrophysiological recording, NMDAR currents are reduced by depolymerized microtubule, which is involved in the transportation of GluN2B-containing vesicles [70]. Likewise, the activation of 5-HT_1A_ receptors can reduce NMDAR currents and target in GluN2B-containing NMDARs, which depends on the microtubule stability [71].

It has been demonstrated that the dendritic specific motor protein KIF17 transports NMDARs along microtubules [72]. KIF17 is a member of kinesin 2 family [45] and is expressed abundantly in mammalian neurons [46]. Overexpressed KIF17 in hippocampal neurons moves dynamically in dendrites at an average velocity of 0.76 *μ*m/sec [69]. Moving KIF17 vesicles comigrate with GluN2B along the dendritic shaft [69]. The colocalization between KIF17 and GluN2B was different with PSD-95-GluN2B or synaptophysin-GluN2B clusters [69]. At least 30% of the GluN2B subunits can be transported from dendrites to synapses by KIF17, which is regulated by transcription factor nuclear respiratory factor 1 (NRF-1) [69, 73]. mLin10 is another PDZ domain containing protein [74, 75]. It colocalizes with KIF17 and GluN2B in dendrites; they coexist as the same large protein complex together with CASK (Lin2) and Veli (Lin7) [72]. In a gliding assay [76], full length KIF17 could carry GluN2B vesicles and move in a plus-end-directed manner on microtubules, but recombinant KIF17 (1-938), lacking the mLin10 interaction domain, failed to move these vesicles on microtubules [72]. These results proved that KIF17 forms complex with mLin10 and GluN2B, and transports the GluN2B-containing vesicles along microtubule.

Intriguingly, this KIF17 regulated NMDAR trafficking undergoes an atypical secretory pathway. Mobile vesicles containing NMDAR subunits GluN1 and GluN2B, KIF17, and postsynaptic adaptor proteins CASK and SAP97 were found to utilize dendritic Golgi outposts for NMDARs trafficking [77]. Knocking down of CASK or SAP97 not only reduced the amount of synaptic NMDARs by 30%–40% but also rerouted NMDARs to the somatic Golgi other than dendritic Golgi outposts [77]. This study suggests not only a mechanism of quick and local turnover of surface NMDARs during synaptic plasticity but also a new route for receptor trafficking.

Moreover, experiments using KIF17 transgenic mice showed enhanced learning and memory. In this transgenic mouse, GluN2B transport in the dendrites was increased, which may result in the enhancement of synaptic plasticity [78]. In KIF17 knockout mice, NMDARs-mediated synaptic currents and plasticity were impaired, along with reduced synaptic GluN2A/2B level and altered GluN2B transport [79]. To explore the mechanisms and physiological significance of KIF17, transgenic mice with different genetic background were generated, including wild-type KIF17 (TgS), KIF17 with S1029A (TgA), or KIF17 with S1029D (TgD) phosphomimic mutations [80]. In TgA/kif17^−/−^ and TgD/kif17^−/−^ mice, synaptic plasticity and spatial memory were both impaired because of insufficient synaptic NMDARs. Thus, the transport of NMDARs regulated by KIF17 is dependent on the phosphorylation [80]. Moreover, the expression of KIF17 coincided with GluN2B transcript expression in the layer III/IV of schizophrenia patients [81]. In summary, KIF17, similar to other adaptor proteins, mediated NMDARs trafficking and synaptic plasticity.

One of the kinesin-5 family protein, KIF11, also named as Eg5, was recently reported to regulate NMDARs surface expression [82]. In this study, two KIF11 inhibitors were used: chemical monastrol and peptide Amyloid beta (A*β*), which directly binds to KIF11 and inhibits its activity by dissociating it from microtubules [83, 84]. Application of A*β* or monastrol on primary neurons led to significant reduction of LTP amplitude [82]. Furthermore, surface labeling revealed a reduction on surface expression level of the NMDAR subunit GluN1 [82]. These results suggest that KIF11 may play a role in LTP through regulating surface NMDARs level, and it could be one of the pathogenesis of A*β*-induced neuronal dysfunction.

## 4. Roles of Kinesin Superfamily Proteins in Kainate Receptors Trafficking

The kainate receptors are composed of different combinations of five subunits, namely, GluR5, GluR6, GluR7, KA1, and KA2 [27]. The localization of GluR5 receptors in the distal dendrites was proved to require the kinesin motor protein KIF17 [31]. When overexpressed KIF17 in neurons, GluR5 receptors were distributed both in the proximal and distal dendrites. However, when neurons were transfected with a dominant negative form of KIF17, the GluR5 receptors were restricted to the proximal dendrites [31]. These results indicate that KIF17 mediates the transport of kainate receptors.

## 5. Roles of Kinesin Superfamily Proteins in GABA_A_ Receptors and GABA_B_ Receptors Trafficking

Many proteins have been identified binding to GABA_A_ receptors and regulate its surface expression, including gephyrin, GABAR-associated protein (GABARAP), protein that links IAP to the cytoskeleton (Plic-1), receptor for activated C-kinase (RACK1), PKC-*β*11, GABA_A_R-interacting factor 1 (GRIF1), Src, AP2, HAP1 (huntingtin associated protein 1), and A-kinase-anchoring protein 150/79 (AKAP150/79) [33, 36, 82, 85–87]. RACK1 binds directly to GABA_A_ receptors at the cell surface. Plic-1 promotes the surface expression of GABA_A_ receptors by stabilizing intracellular GABA_A_ receptors from proteasome degradation [85].

HAP1 (huntingtin associated protein 1) interacts with GABA_A_ receptors directly and modulates synaptic GABA_A_ receptors by inhibiting receptor degradation and facilitating receptor recycling [36]. HAP1 was later identified as an adaptor that directly binds to KIF5 and links KIF5 to GABA_A_ receptors [88]. KIF5 is colocalized with GABA_A_ receptors at dendrites and is frequently seen associated with vesicles close to the postsynaptic membrane. The HAP1-KIF5 complex could mediate insertion of GABA_A_ receptors into the surface membrane at synapses [88]. By altering the expression level of HAP1, the GABA_A_R vesicle movement through dendrites was affected [88]. When the HAP1 binding domain (HBD) of KIF5B was overexpressed in neurons, the surface level of GABA_A_ receptors had a significant decrease. The mIPSC amplitude also showed a significant difference by overexpressing the HAP1-HBD domain. These results suggest that the delivery of GABA_A_ receptors from internal compartments to surface and synaptic sites is dependent on HAP1-KIF5 motor protein complex. In KIF5A conditional knockout mice, GABA_A_R transmission was impaired following significantly reduced mean amplitude of mIPSCs and less GABA_A_R puncta was found distributed in the dendrites of hippocampal neurons [89]. KIF5A specifically interacts with GABA_A_R-associated protein (GABARAP) which is known to be involved in GABA_A_R trafficking. Disrupting the KIF5A-GABARAP interaction significantly reduced the surface GABA_A_R expression in neurons [89]. Taken together, both KIF5/HAP1 and KIF5A/GABARAP complexes are important for surface and synaptic localization of GABA_A_Rs.

In addition, KIF5A was further verified to interact with GABA_A_R-interacting factor 1 (GRIF1), a novel GABA_A_Rs interacting protein [33],* in vivo* in brain tissues [37], which could be another possible regulatory machinery in GABA_A_ receptors trafficking.

It has been recently reported that KIF5C determines axonal localization of GABA_B_R1a, one of the most abundant isoforms of GABA_B_R subunit GABA_B_R1 [90]. When hippocampal neurons were transfected with myc-GABA_B_R1a and RFP-KIF5C-DN (dominant negative form of KIF5C), the axonal targeting of GABA_B_R1a was markedly reduced compared to control [91]. It is interesting to notice that, GABA_B_R heteromers containing GABA_B_R1a are expressed axonal and somatodendritic, while those containing another GABA_B_R1 isoform GABA_B_R1b are exclusively located in the somatodendritic domain [92]. Therefore, KIF5 family protein may also regulates the axonal localization of GABARs in an isoform-specific way.

KIF21B, another kinesin family protein, is expressed dominantly in brain, eye, and spleen. It is enriched in dendrites and growth cones at neurite tips [95, 96]. KIF21B forms complex with GABA_A_ receptors and GABA_A_ receptor-associated protein (GABARAP) [94]. In cultured hippocampal neurons, KIF21B is colocalized with GABA_A_ receptors in dendrites and soma, and knockdown of KIF21B caused a reduction of GABA_A_ receptor number in the cell surface [94]. We could see that KIF21B may participate in the delivery of GABA_A_ receptor transport along dendrites.

## 6. Conclusions and Discussion

Glutamate receptors and GABA receptors trafficking play an important role in synaptic plasticity. NMDA receptors (NMDARs) are the main triggers for the induction of LTP and LTD, and their activation is tightly regulated by the activation of GABA receptors. AMPA receptors mediate the synaptic response and their trafficking is believed to be an important target of modulation during synaptic plasticity.

**Figure 1:**
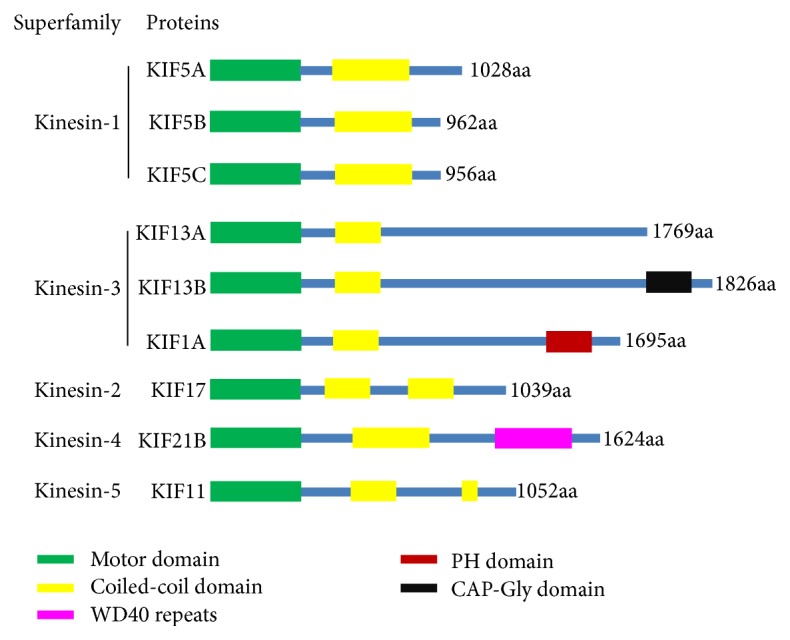
Domain structures of kinesin superfamily proteins. The domain structure of the kinesin family proteins discussed in this review is provided in cartoon diagram. The protein length information is indicated after each diagram, and the name of each single domain is indicated.

In our review, we described the relationship between some kinesin superfamily proteins and synaptic plasticity. It is suggested that KIF5B can transport AMPARs by the direct interaction with GRIP1. KIF1A could form a complex with AMPA receptor-GRIP complex through liprin-*α* and may also regulate AMPAR trafficking. In* C. elegans*, unc116/KIF5 and KLP-4/KIF13 also regulate the trafficking of GLR-1 glutamate receptors. KIF17 contributes to NMDARs trafficking by affecting GluN2B subunit through the dendritic Golgi outposts; KIF11/Eg5 is related to the trafficking of NMDA/GluN1 receptors. KIF5A, KIF5C, and KIF21B can modulate the delivery of GABA receptors. But the mechanism underlying the specificities between kinesin superfamily and the cargo receptors remains unknown. According to their domain structures, all of the summarized KIFs are N-kinesin proteins as their motor domains reside in the N-terminus (Figure 1). The sequences of the kinesin motor domain are very similar. Especially for KIF5 proteins, KIF5A was found to have specific regulatory role for GABA_A_Rs while KIF5B prefers AMPARs [52, 89]. It would be interesting to look at the detailed protein sequences on other domains to search for the kinesins' cargo specificity (Figure 1).

The number of identified kinesin superfamily proteins has been increasing. It is possible that, except KIF5, KIF17, KLP4/KIF13, KIF1A, KIF11, and KIF21B, more kinesin proteins participate in the receptor trafficking and synaptic plasticity.

In summary, the kinesin superfamily proteins, along with a lot of adaptor proteins, participate in the synaptic plasticity by regulating the trafficking of the ion-channel receptors. The different structure of the KIFs may also play important roles during this process (Figure 1 and Table 1).

## Figures and Tables

**Table 1:** Neuroreceptors transported by kinesin superfamily proteins (KIFs).

Motor proteins	Superfamily	Neuroreceptors	Interactome	Transport	References
KIF5	Kinesin-1	AMPARs (GluA2)	GRIP1	Dendritic	[19, 52]
unc116/KIF5	Kinesin-1	GLR-1	Unknown	Unknown	[58]^∗^
KLP-4/KIF13	Kinesin-3	GLR-1	Unknown	Unknown	[61]^∗^
KIF1A	Kinesin-3	AMPARs (GluA2/3)	liprin-*α*	Axonal and Dendritic	[59, 60]^#^
KIF17	Kinesin-2	KARs (GluR5)	Undefined	Dendritic	[31]
KIF17	Kinesin-2	NMDARs (GluN2B)	m-Lin10 (Mint1, X11) m-Lin2 (CASK) m-Lin7 (MALS, Velis) SAP97	Dendritic	[69, 72, 77, 93]
KIF11/Eg5	Kinesin-5	NMDARs (GluN1)	Undefined	Undefined	[84]
KIF5	Kinesin-1	GABA_A_Rs	HAP1	Dendritic	[88]
KIF5A	Kinesin-1	GABA_A_Rs	GABARAP	Dendritic	[89]
KIF21B	Kinesin-4	GABA_A_Rs	GABARAP	Dendritic	[94]
KIF5C	Kinesin-1	GABA_B_R1a	Undefined	Axonal	[91]

Indication: ^∗^the regulatory role of KIF protein on neuroreceptors trafficking was found in *C. elegans*, while others were studied in mammalian cells.  ^#^The regulatory role of KIF1A on AMPAR trafficking was deduced from previous studies. There is no direct evidence on whether KIF1A could regulate AMPARs transport in axons and dendrites up till now.
